# Post-COVID depression and its multiple factors, does Favipiravir have a protective effect? A longitudinal study of indonesia COVID-19 patients

**DOI:** 10.1371/journal.pone.0279184

**Published:** 2022-12-30

**Authors:** Bumi Herman, Andrea Bruni, Ekachaeryanti Zain, Azhar Dzulhadj, Aye Chan Oo, Viwattanakulvanid

**Affiliations:** 1 College of Public Health Science Chulalongkorn University Thailand, Bangkok, Thailand; 2 Faculty of Medicine, Hasanuddin University, Makassar, Indonesia; 3 Mental Health Officer, World Health Organization, Baghdad, Iraq; 4 Department of Psychiatry, Graduate School of Medicine, Niigata University, Niigata, Japan; 5 Department of Psychiatry, Faculty of Medicine, Mulawarman University, Samarinda, Indonesia; 6 School of Biomedical Science, The University of Western Australia, Perth, Australia; Universidade Federal do Rio Grande do Sul, BRAZIL

## Abstract

**Background:**

Coronavirus disease (COVID-19) not only has a long-term effect on its survivors, it also affects their quality of life, including inducing depression as a possible manifestation of central nervous system disruption. Favipiravir shows promising efficacy as an antiviral drug for the treatment of COVID-19. However, its effect on the sequelae of COVID-19 has not been explored. Therefore, this study aims to assess the impact of Favipiravir and address the factors associated with post-COVID depression in Indonesia.

**Method:**

This cohort study conducted a post-COVID-19 survey on Indonesian patients who were diagnosed by using real-time polymerase chain reaction (RT-PCR) and antigen tests until January 2022. An online questionnaire was distributed to obtain information on demographics, comorbidities, health behavior, symptoms, and treatment. The propensity technique was used to allocate the participants into the favipiravir and nonrecipient groups (1:1). The Patient Health Questionnaire-9 (PHQ-9) was used for outcome measurement. The cohort was followed up biweekly for 60 days after onset/diagnosis to determine the occurrence of depression. Cox regression analysis with an adjusted odds ratio and 95% confidence interval was used to estimate the effect of favipiravir on post-COVID-19 depression.

**Results:**

The data included the information of 712 participants, of whom 18.54% had depression within 60 days after onset/diagnosis. Depression was higher in the nonrecipient group (21.06%) than in the favipiravir group (16.01%). After adjustment by other factors, favipiravir prescription was found to be associated with depression (aOR 0.488, 95% CI 0.339–0.701 p < 0.001). In accordance with the PHQ-9 subset, favipiravir exerted a significant protective effect against depressive mood and loss of interest. However, patients living alone were prone to experiencing loss of interest (aOR 2.253, 95% CI 1.329–3.818, p = 0.003).

**Conclusion:**

The data obtained in this preliminary survey suggested that favipiravir may be useful for preventing post-COVID depression. However, further study is needed. Moreover, the provision of mental health support, particularly to those who live alone, must be ensured.

**Trial registration:**

Registry NCT05060562.

## Introduction

### Epidemiology

Coronavirus disease 19 (COVID-19), which is caused by the severe acute respiratory syndrome coronavirus 2 (SARS-CoV-2), is a significant public health concern given that a total of 364 million cases of this disease with 5.6 million mortalities were recorded in January 2022. In Southeast Asia, 51 million people have been infected with SARS-CoV-2, resulting in 733,767 fatalities. Indonesia recorded the highest cases in the Southeast Asian region, accounting for 4.3 million diagnoses and 144,261 mortalities [1].

The clinical manifestations of COVID-19 are dependent on comorbidities, particularly chronic diseases, such as hypertension [2]. However, the rapid mutation of this virus puts all people at similar risk. Furthermore, emerging new variants (such as the omicron variant, which is characterized by its rapid transmission and high infectivity), have also been known to affect nonhealth aspects. Therefore, travel restrictions and strict health protocols are still needed to curb the staggering infection of COVID-19. COVID-19 is no longer a respiratory disease that presents with cough or fever [3]. Instead, it manifests as a systemic disease. At its later stage, patients may still experience its sequelae.

### Defining the sequelae of COVID-19

Persistent symptoms after a COVID-19 episode affect quality of life particularly in the first month after recovery. Regardless of the type of treatment, approximately 30%–50% of COVID survivors continue to experience symptoms a month after COVID-19 [4, 5]. Given that a number of people have reported specific symptoms after COVID-19, this collection of symptoms must be established as a syndrome associated with COVID-19. The National Institute for Health and Care Excellence, together with the Scottish Intercollegiate Guidelines Network and the Royal College of General Practitioners, introduced a formal definition for long COVID as “signs and symptoms developed during or following a disease consistent with COVID-19 and which continue for more than 4 weeks but they are not explained by alternative diagnosis” [6].

Post-COVID symptoms have also been defined by other time-specific criteria. For example, some people might be asymptomatic in most cases, whereas others require hospitalization. Therefore, providing a uniform definition of post-COVID-19 syndrome is challenging. One author introduced the post-COVID time measurement to the onset or after hospital discharge, wherein the transition phase occurs at approximately 4–5 weeks. The following stages after the transition phase include acute post-COVID (5–12 weeks), long post-COVID symptoms (12–24 weeks), and persistent post-COVID symptoms (more than 24 weeks) [7].

Depression occurred as the residual symptom of COVID-19 in 12% of COVID-19 cases followed up for 3 months [8]. Depression could occur as early as the time of infection and may concurrently present with anxiety and stress [9]. Psychiatric changes are hypothetically linked to neurological changes and may persist for several months. The ascending infection theory, alterations in the supporting cells of the neuron and adjacent nerve tissue [10], disruption of the blood-brain barrier (BBB) following systemic inflammation [11], and microvascular damage and hypoxic events [12] are suggested to be the possible underlying mechanisms of these changes. Moreover, the disruption of tryptophan absorption and metabolism [13] in the gastrointestinal tract in patients with COVID may serve as an indirect mechanism of depression [14, 15] and is mainly expressed as central fatigue and memory disturbance [16].

Given that the quality of life of post-COVID patients highly deteriorates due to depression, many studies have encouraged the importance of addressing depression and post-traumatic stress disorder. In Indonesia, depression due to COVID-19 was mainly assessed in healthcare workers and the general population. However, studies on depression in the post-COVID period remain limited, and the magnitude of this sequelae has not been described comprehensively.

### Favipiravir

This antiviral drug disrupts RNA viral replication via RNA polymerase activity [17]. As a result, favipiravir reduces the viral shedding duration of SARS-COV2 and the level of proinflammatory markers [18]. The initial dose of this drug for the treatment of COVID-19 is 1600 mg every 12 h for the first day then 600 mg twice daily. This dose is recommended for influenza. Several trials and current guidelines in other countries have introduced higher doses (1800 mg twice daily for loading doses followed by 800 mg twice daily) [19]. However, the results of different doses remain inconclusive. Nevertheless, favipiravir remains superior in terms of its mechanism of action relative to other antiviruses, such as oseltamivir [20], particularly in mild-to-moderate cases.

How does favipiravir reduce subsequent neuropsychiatric changes, including depression? A study in China revealed the relationship between prolonged viral shedding and proinflammatory response [21]. Therefore, the administration of favipiravir reduces the number of viruses and attenuates proinflammatory conditions, particularly in the central nervous system (CNS), precluding the occurrence of CNS sequelae. However, the effects of favipiravir on brain function, including its direct impact on neurotransmitters, its ability to function as a neuroprotector, or even its capability to enhance neuroplasticity in patients with COVID-19, lack proof.

### Objective and hypothesis

This study aims to address the impact of favipiravir in preventing post-COVID depression and concurrently evaluate other plausible factors. We hypothesized that taking favipiravir will benefit reducing clinical depression after COVID by preventing CNS injury and neurotransmitter pathway disruption.

## Methodology

### Setting and study design

**Fig 1:**
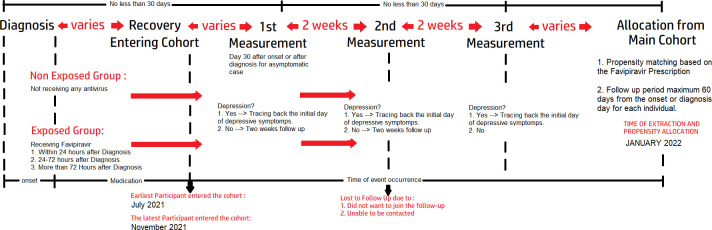
Timeline of the cohort. Time of participant recruitment and allocation to create a quasi-experimental design.

We utilized the Indonesian POST-COVID survey data that started in July 2021. The data consisted of the clinical and demographic information of patients with COVID-19 from all provinces in Indonesia that were submitted through the online link. We expanded the accessibility of the link to several COVID survivor groups and other social media platforms. We relied on the snowball approach to obtain high participation. The participants who were included in this study provided their baseline demographic and clinical status during their COVID-19 episode. Supporting information was obtained from the participants with their permission and included medical resumes from the hospital and independent lab assessments, which were requested by the participants as part of their COVID-19 routine care. At the beginning of the submission, formal permission to participate in consecutive clinical follow-ups related to various post-COVID symptoms were offered in written form. In addition, the participants who agreed to undergo follow-up were contacted in accordance with the specific measurement time. After the cohort reached a considerable number of participants, we sorted the participants on the basis of a specific outcome. For this study, we selected the participants who entered the cohort until November 2021 and were followed up until January 2022. Fig 1 illustrates the timeline of data collection.

### Participant eligibility

The inclusion criteria in this study consisted of age, case definition, and recovery status. The eligibility criteria did not include a minimum age as long as the participants could provide their information by using the tool. Furthermore, the participants should fulfill the definition of a COVID-19 case, that is, COVID-19 diagnosed through the collection of nasopharyngeal samples and confirmed by using real-time polymerase chain reaction (RT-PCR) or other nucleic acid amplification tests [22] because these tests exhibit high sensitivity and specificity in the detection of SARS COV2 in nasopharyngeal samples [23]. An antigen test was also acceptable when performed in line with the national guidelines, that is, diagnosis is performed on a collected nasopharyngeal or anterior nasal sample with the sensitivity of 80% and specificity of at least 97% [24]. Individuals who tested positive for IgM antibodies (indicating the acute phase) should undergo RT-PCR or antigen test for confirmation. In accordance with the inclusion criteria of the primary cohort, the participants were deemed eligible to enter the cohort after their recovery was declared by either a physician or in accordance with any other acceptable guidelines. The participants may end home isolation on the basis of symptom improvement. This included [1] 10 days for asymptomatic cases or [2] 10 days after the onset plus 3 days without fever and respiratory symptoms in accordance with the guidelines of the World Health Organization [25]. Patient discharge confirmed by RT-PCR results was also acceptable, particularly in hospitalized individuals who had been granted special considerations by the physician.

The exclusion criteria in this study consisted of participant responses and specific variables. Participants should have recovered within 30 days of onset/diagnosis. Participants who were unable to be contacted or refused to provide further information were excluded from data extraction. We also excluded those who were suspected to have SARS-COV2 reinfection or repositivity history based on self-reported information. Individuals who received other antiviral medications were also excluded. However, participants who suffered from all comorbidities, including those with self-reported mental health disorders, were still included in the study.

### Variables

We collected three types of data. First, the demographic data included age at diagnosis, sex at birth, occupation (classified into binary responses: medical personnel and nonmedical personnel), level of education, island of origin, type of living area (from rural to urban), living companion, social activity, and the possession of health insurance.

The next set of data included the factors related to current health status and comorbidities, including body mass index (body weight in kilograms over the square of body height in meters) at the time of infection, health behavior (including smoking and alcohol drinking within 3 months before diagnosis and moderate physical activity (30–40 min of physical activity involving a warm-up, main session, and cool-down), as well as the presence of other diseases and current treatment status (controlled or uncontrolled).

Finally, we obtained clinical data related to COVID-19 symptoms along with the treatment received by the participants. These data consisted of the time and method of diagnosis confirmation, the signs and total duration of the symptoms, prescribed medication, advanced and intensive care (oxygen supplementation or plasma during convalescence) as well as the unit of care (home isolation, hospitalization, or referred cases), and vaccination status during infection.

### Tools

We developed the questionnaire on the basis of the possible long-term effects of COVID-19 and its plausible factors. SARS-COV2 detection via RT-PCR was based on the discovery of ORF1-ab, nucleocapsid, RNA-dependent RNA polymerase (RdRP), or Spike genes associated with SARS-COV2 infectivity [26]. All tools for diagnosis, including the antigen tests/lateral flow as recommended by the WHO, underwent an approval procedure by the Indonesian Food and Drug Administration.

### Exposure

Our object of interest is the administration of the favipiravir regimen. In accordance with Indonesia’s COVID-19 guidelines, 1600 mg of loading doses are given orally every 12 h on the first day, followed by 600 mg every 12 h from day 2–5 [27]. In practice, this regimen is initiated in those with mild symptoms and more severe cases that can take medicine via the oral route. This recommended dose is lower than that in Thailand [28] but similar to that in Japan [29]. Favipiravir was prescribed on the basis of the judgment of the physician and was mainly given to those without renal impairment [30] and to individuals who were unlikely to experience severe side effects, including pregnant women [31] and hypersensitive individuals. In addition, the prescription could also be made on the basis of telemedicine assessment or on-site examination for eligible home-isolated patients. We compared this group with the group of individuals whose disease resolved entirely without taking any other antiviral medications.

### Outcomes

Depression based on screening is the outcome of this study. We implemented depression screening by using the self-reported Patient Health Questionnaire 9 (PHQ-9), which represents the situation over the past 2 weeks [32]. Furthermore, we collected the symptoms of depression in accordance with Diagnosis and Statistical Manual of Mental Disorders (DSM-5) to strengthen the results of PHQ-9 [33]. PHQ-9 is widely used in studies related to COVID [34] and is strongly correlated with the structural clinical interview for depression in psychiatric centers [35]. This questionnaire consists of nine Likert-scale questions with scores ranging from 0 to 3 per question with a total score ranging from 0 t0 27. A score above 4 could indicate depression with a sensitivity of 91% albeit with low specificity (60%) in a study of 580 people [32]. This tool is also valid for patients with respiratory disorders [36]. We collected the outcomes for a 2-week interval until suggestive depression occurred, and we did not repeat the measurement on those who developed depression. An additional question was asked to confirm the day when the depressive symptoms began: "How many days from today did you begin to experience these symptoms?” For asymptomatic patients, the first data collection was conducted on day 30 after the onset or day of diagnosis. The total follow-up period was 60 days and would cover the post-COVID period.

### Study size and possible bias

We noticed that the involvement of all eligible participants from the primary cohort would introduce bias, particularly unequal allocation. Therefore, we performed propensity match selection and allocated the participants equally to obtain an unbiased cohort. The factors selected for matching were age, sex, occupation, island of origin, self-reported allergy, and hypersensitivity because these factors were linked to favipiravir distribution and access, as well as a general contraindication for favipiravir prescription. We selected the cases on the basis of exact or fuzzy matching with the match tolerance of 0.05 for each propensity score of exposed and nonexposed cases.

We estimated the sample size with 5% type I error, 80% power, equal allocation between two groups (1:1), and the assumption that favipiravir exerts a superior effect on post-COVID depression. In addition, we assumed that the difference in the proportion of depression between the exposed and nonexposed cases was 7.5%. Hence, a sample size of 348 was required for each group with a total of 696 participants.

We acknowledged potential biases that remained after the adjustment to obtain an unbiased subset from the primary cohort, which might represent the actual situation of COVID. Recall bias, particularly in the data of the symptoms, was inevitable, especially for people who underwent home isolation. However, telemedicine could ensure the reliability of the symptoms because home isolation required patients to observe and record their symptoms. We emphasized this issue in the consent form by stating that participants should highly rely on their observation chart when filling in the form. We noted different periods of the favipiravir prescription that should be accommodated to prevent statistical bias if favipiravir was treated as a time-fixed covariate. However, we acknowledged that the prescription day would not always represent the time that the medicine was ingested.

### Quantitative variables

We calculated body mass index by using the formula of body weight in kilograms over the square of height in meters and performed no further classification. In accordance with the cutoff, as mentioned earlier, we categorized PHQ-9 into a binary variable (depression and no depression) by using a cutoff of 4. No discretization was made for other continuous data.

### Statistical analysis

Given that the nature of the baseline questionnaire was set to collect a complete response for submission, no data imputation or other technique to handle missing baseline data was needed. We performed descriptive analysis and normality tests to assess and determine the distribution of continuous data, followed by bivariate analysis by using the χ^2^, Fisher’s exact, Mann–Whitney, and Kruskal–Wallis tests to address the similarity of variables between the groups. Testing for multicollinearity among predictors was crucial considering that further tests were based on regression. Variables with possible collinearity were subjected to bivariate analysis, and we selected the factor that could represent other factors for inclusion in the final analysis on the basis of collinearity analysis. We assumed that the duration of shortness of breath was associated with the duration of other symptoms and could represent disease severity.

Several variables were assumed to possibly interact as moderators or mediators, demonstrating a possible indirect association. Sobel test and logistic regression were conducted to assess this indirect effect. This study assumed that the severity of the disease mediates the effect of favipiravir on depression. Furthermore, several variables, such as sociodemographic factors, might act as a moderator. We implemented a similar mediation analysis to rule out the indirect effect of other medications on depression.

For the final analysis, we applied Cox regression with other confounders (moderators and mediators) to identify the adjusted effect of favipiravir on post-COVID depression. The risk was presented with a 95% confidence interval. Furthermore, we performed symptom-specific analysis (loss of interest, depressive mood, sleep disorders, concentration disturbance, and fatigue) with Cox regression and provided Kaplan–Meier curves.

### Ethical approval

We obtained approval from the Research Ethics Review Committee for Research Involving Human Research Participants, Faculty of Medicine, Hasanuddin University (full-board review number 758/UN4.6.4.5.31/PP36/2021). Participants provided written consent when they submitted the data to the cohort. The online submission system allowed all parties to store the submitted data. Given that this study did not include minors, no consent from guardians was required. We deidentified, stored, and utilized the data accordingly to ensure confidentiality. Any case that required immediate treatment was referred to specialist care. This research is part of the study registered under the clinicaltrials.gov number NCT05060562.

## Results

### Baseline characteristic

**Fig 2:**
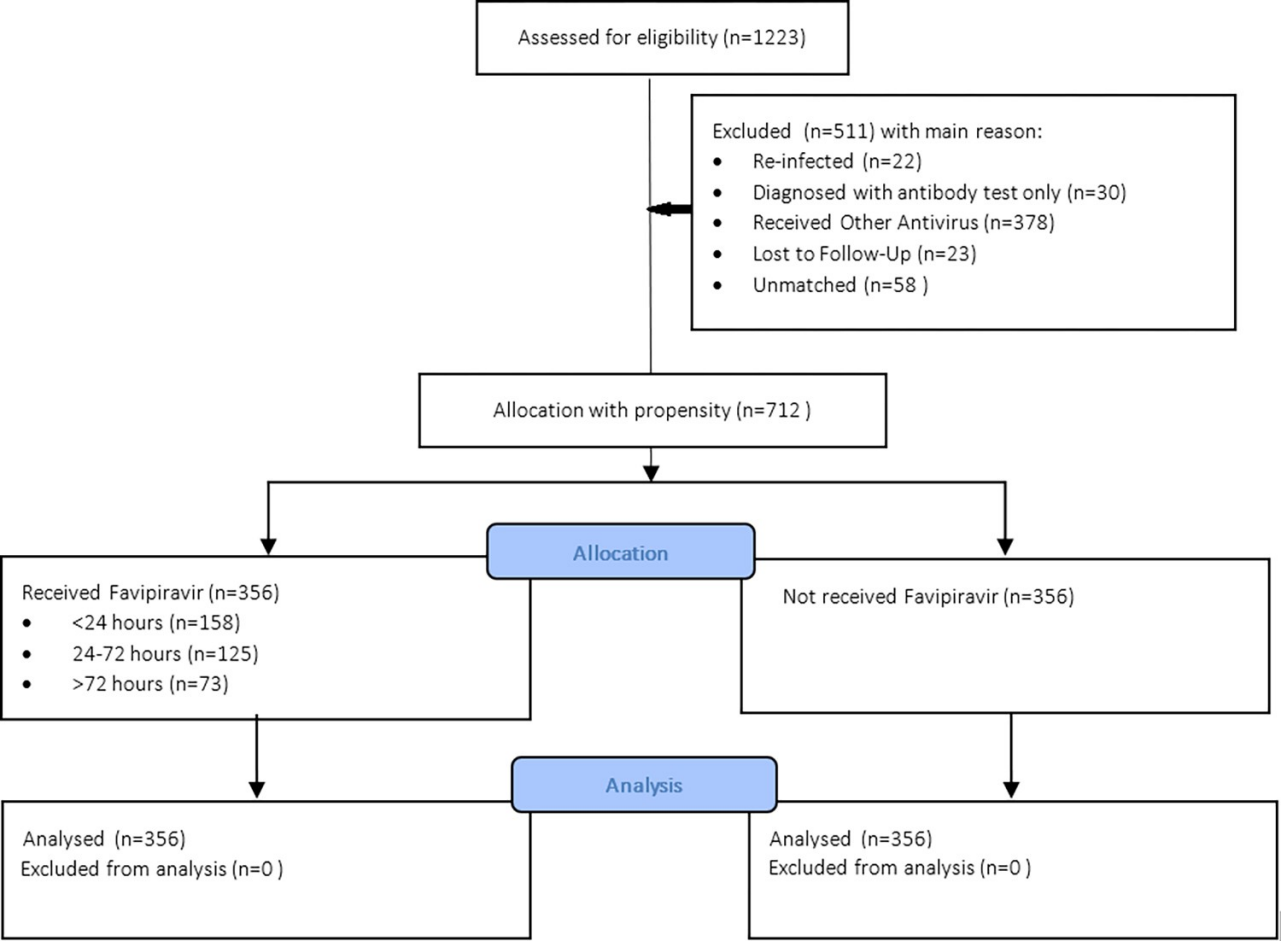
Participant chart.

We extracted 712 participants from the primary cohort and allocated them following propensity matching. A total of 323 (45.4%) participants were diagnosed solely by using RT-PCR, and 175 (24.6%) underwent RT-PCR confirmation after antigen or antibody tests. A total of 203 (28.5%) participants were screened by using antigen tests only, and 11 (1.5%) were retested by antigen testing after showing positive antibody test results. Among those who received favipiravir (exposed group), 158 (44.4%) received the regimen <24 h after diagnosis, 125 (35.1%) received it within 24–72 h, and 73 (20.5%) participants took the pills >72 h. The number of people with depression within 60 days of follow-up was 75 (21.06%) in the nonrecipient group, whereas only 57 people (16.01%) in the favipiravir group developed depression. The overall prevalence of depression in this cohort was higher than that in the meta-analysis (132/756 or 18.54%). However, the highest PHQ-9 score was 9, which indicated mild depression. Fig 2 depicts the selection of participants from the primary cohort until the follow-up.

Table 1 presents the participants’ demographic and risk factor assessments extracted from the cohort. Despite matching by sex (p = 0.018) and island (p < 0.001), these demographic factors significantly differed between groups. Furthermore, the percentage of people who lived alone and received favipiravir was higher than that of nonreceivers, whereas people who lived with their family or relatives tended not to receive favipiravir (p < 0.005). A similar pattern was seen in the social activity variable, wherein those who worked or studied outside their homes were likely to receive favipiravir but not those who stayed at home (p = 0.003). In addition, the mean age was higher in the favipiravir group (p < 0.001) than in the other group. The disparity in treatment was seen in occupation factors, wherein medical personnel had a higher percentage of accessing favipiravir (55.2%) than nonmedical personnel (47.8%), although this disparity was not significant. Other factors possibly linked to depression, such as educational level, type of living area, and health insurance coverage, did not significantly differ between groups (p > 0.05).

**Table 1:** Demographic characteristics of participants (n = 712).

Variables	Subset	No favipiravir (%)	Favipiravir (%)	p-value
Sex	Female	228 (53.6)	197 (46.4)	0.018
	Male	128 (44.6)	159 (55.4)	
Occupation	Nonmedical personnel	262 (52.2)	240 (47.8)	0.071
	Medical personnel	94 (44.8)	116 (55.2)	
Educational level	Up to diploma	182 (49.6)	185 (50.4)	0.822
	Bachelor’s degree and graduate Level	174 (50.4)	171 (49.6)	
Island	Sumatera	50 (43.1)	66 (56.9)	<0.001
	Jawa	148 (62.2)	90 (37.8)	
	Bali and Lesser Sunda	23 (41.1)	33 (58.9)	
	Kalimantan	69 (43.9)	88 (56.1)	
	Sulawesi	61 (47.7)	67 (52.3)	
	Maluku and Papua	5 (29.4)	12 (70.6)	
Type of living area	Rural area	90 (48.6)	95 (51.4)	0.754
	Urban not capital	94 (48.7)	99 (51.3)	
	Capital area	172 (51.5)	162 (48.5)	
Living with	With family, colleagues, or relatives	242 (54.0)	206 (46.0)	0.005
	Alone	114 (43.2)	150 (56.8)	
Social activity (work and study)	Shifted to home-based activities	180 (56.3)	140 (43.8)	0.003
	Outside home, most of the time	176 (44.9)	216 (55.1)	
Covered by health insurance	National health insurance	180 (51.5)	177 (48.5)	0.410
	Private insurance	75 (45.5)	90 (54.5)	
	No Insurance or inactive	93 (51.1)	89 (48.9)	
Age	Mean ± std deviation	31.02 ± 9.99	33.66 ± 10.18	<0.001
BMI	Mean ± std deviation	23.73 ± 4.60	23.88 ± 4.92	0.172
Hypertension	No/unknown	334 (50.5)	327 (49.5)	0.437
	Yes, controlled	12 (38.7)	19 (61.3)	
	Yes, uncontrolled	10 (50.0)	10 (50.0)	
Diabetes mellitus	No/unknown	341 (49.8)	344 (50.2)	0.100
	Yes, controlled	11 (73.3)	4 (26.7)	
	Yes, uncontrolled	4 (33.3)	8 (66.7)	
Asthma	No/unknown	338 (49.4)	346 (50.6)	0.089^
	Yes, controlled	14 (58.3)	10 (41.7)	
	Yes, uncontrolled	4 (100)	0 (0)	
HIV	No/unknown	355 (49.9)	356 (50.1)	1.000^
	Yes, controlled (undetectable)	1 (100)	0 (0)	
COPD	No/unknown	352 (49.9)	353 (50.1)	1.000^
	Yes, controlled	3 (50)	3 (50)	
	Yes, uncontrolled	1 (100)	0 (0)	
Cancer/malignancy	No/unknown	353 (49.8)	356 (50.2)	0.249^
	Yes	3 (100)	0 (0)	
Dyspepsia syndrome	No/unknown	213 (51)	205 (49)	0.543
	Yes	81 (47.1)	91 (52.9)	
Hypersensitivity/allergy	No/unknown	292 (47.6)	322 (52.4)	0.001
	Yes	64 (65.3)	34 (34.7)	
Heart disease	No/unknown	350 (49.9)	352 (50.1)	0.524
	Yes	6 (60)	4 (40)	
Stroke	No/unknown	355 (50)	355 (50)	1.000^
	Yes	1 (50)	1 (50)	
Mental health disorder	No/unknown	343 (49.7)	347 (50.3)	0.386
	Yes	13 (59.1)	9 (40.9)	
Autoimmune disease	No/unknown	354 (50.2)	351 (49.8)	0.451^
	Yes	2 (28.6)	5 (71.4)	
Kidney disease	No/unknown	353 (49.8)	356 (50.2)	0.249^
	Yes	3 (100)	0 (0)	
Liver disease	No/unknown	353 (49.9)	355 (50.1)	0.624^
	Yes	3 (75)	1 (25)	
Gastrointestinal bleeding	No/unknown	355 (50.1)	354 (49.9)	1.000^
	Yes	1 (33.3)	2 (66.7)	
Smoking Status	Never	326 (50.4)	321 (49.6)	0.515
	ever/currently Smoking	30 (46.2)	35 (53.8)	
Drink alcohol at least three months before diagnosis	No	331 (49.3)	341 (50.7)	0.104
	Yes	25 (62.5)	15 (37.5)	
Frequency of doing moderate exercise in a week before diagnosis	>3 times per week	39 (58.2)	28 (41.8)	0.277
	2–3 times per week	49 (45.8)	58 (54.2)	
	One or less per week	268 (49.8)	270 (50.2)	
Infected more than 14 days after receiving the second dose of vaccine?	No	252 (47.5)	278 (52.5)	0.508
	Yes	104 (57.1)	78 (42.9)	

^Fischer’s exact test. Other categorical variables were tested with χ^2^ test. Finally, all continuous data were tested with Mann–Whitney.

As for comorbidities, people with a history of allergy were not prescribed favipiravir as much as those without a history of allergies (p < 0.001). No significant difference was found between these groups regarding other diseases (including self-reported mental health disorders) and health behavior (p > 0.05). Favipiravir was less prescribed among those who were fully vaccinated for more than 14 days (hence, considered as protected individuals) although it was not significantly different (p = 0.508).

A significant difference in symptoms (p < 0.001 except for diarrhea, p = 0.03) and treatment variables was found between the groups as presented in Table 2. Except for runny nose, for which the nonexposed group had a longer duration (p = 0.015), symptoms lasted longer in those who received favipiravir. Other medications, except for vitamin C (p = 0.105) and other antibiotics (p = 0.535), were prescribed at significantly higher rates in people who received favipiravir. More than 60% of patients in the favipiravir group received oxygen supplementation (p = 0.034). Favipiravir administration was also frequent in hospitalized cases or cases that were referred to the hospital after home isolation (p = 0.004). However, the groups had no significant differences in intensive care rate (p = 0.124) or convalescent plasma administration (p = 0.056). Bivariate association revealed a nonsignificant difference in depression between the two groups after 60 days of follow-up (crude OR 0.71 95% CI 0.49–1.05 p = 0.089).

**Table 2:** Symptoms and treatment received by participants during their COVID episode.

Variables	Subset	No favipiravir (%)	Favipiravir (%)	p-value
Fever	Mean ± std deviation	1.94 ± 1.67	2.26 ± 1.13	<0.001
Cough	Mean ± std deviation	2.79 ± 3.15	3.47 ± 2.55	<0.001
Shortness of breath	Mean ± std deviation	1.10 ± 1.53	1.42 ± 1.12	<0.001
Myalgia and fatigue	Mean ± std deviation	1.69 ± 1.91	2.02 ± 1.42	<0.001
Headache	Mean ± std deviation	1.77 ± 1.95	2.11 ± 1.46	<0.001
Anosmia/loss of smell	Mean ± std deviation	2.12 ± 2.68	2.15 ± 1.66	<0.001
Ageusia/loss of taste	Mean ± std deviation	1.70 ± 2.32	1.79 ± 1.51	<0.001
Diarrhea	Mean ± std deviation	0.55 ± 0.98	0.61 ± 0.83	0.03
Runny nose	Mean ± std deviation	1.10 ± 1.72	1.04 ± 1.10	0.015
Insomnia	Mean ± std deviation	1.79 ± 2.08	2.35 ± 1.922	<0.001
Days of treatment	Mean ± std deviation	14.09 ± 4.18	15.34 ± 3.48	<0.001
Received an antipyretic?	No fever	43 (86)	7 (14)	<0.001
	<24 h after the fever appeared	165 (44.6)	205 (55.4)	
	24–72 h after the fever appear	60 (46.9)	68 (53.1)	
	>72 h after the fever appear	18 (51.4)	17 (48.6)	
	Not receiving any treatment	34 (47.2)	38 (52.8)	
Received azithromycin after diagnosis	<24 h	82 (35.8)	147 (64.2)	<0.001
	24–72 h	75 (37.7)	124 (62.3)	
	>72 h	42 (45.2)	51 (54.8)	
	Not receiving any treatment	157 (82.2)	34 (17.8)	
Received any anti-inflammatory after diagnosis	<24 h	61 (37.0)	104 (63.0)	<0.001
	24–72 h	28 (37.3)	47 (62.7)	
	>72 h	21 (51.2)	20 (48.8)	
	Not receiving any treatment	246 (57.1)	185 (42.9)	
Received any medicine for cough relief after diagnosis	<24 h	109 (48.9)	114 (51.1)	<0.001
	24–72 h	71 (36.2)	125 (63.8)	
	>72 h	39 (47.0)	44 (53.0)	
	Not receiving any treatment	137 (65.2)	73 (34.8)	
Received vitamin C daily at doses higher than 500 mg after diagnosis	<24 h	163 (48.5)	173 (51.5)	0.105
	24–72 h	78 (45.9)	92 (54.1)	
	>72 h	52 (51.0)	50 (49.0)	
	Not receiving any treatment	25 (56.8)	19 (43.2)	
Received vitamin D daily at doses higher than 400 IU after diagnosis	<24 h	124 (44.8)	153 (55.2)	0.017
	24–72 h	60 (53.1)	53 (46.9)	
	>72 h	70 (46.1)	82 (53.9)	
	Not receiving any treatment	36 (54.5)	30 (45.5)	
Received zinc at doses of at least 20 mg after diagnosis	<24 h	99 (43.0)	131 (57.0)	<0.001
	24–72 h	59 (39.6)	90 (60.4)	
	>72 h	37 (56.9)	28 (43.1)	
	Not receiving any treatment	71 (50.7)	69 (49.3)	
Received another antibiotic after diagnosis	<24 h	17 (63.0)	10 (37.0)	0.535
	24–72 h	7 (43.8)	9 (56.3)	
	>72 h	8 (53.3)	7 (46.7)	
	Not receiving any treatment	324 (49.5)	330 (50.5)	
Received oxygen supplementation	No	330 (51.6)	309 (48.4)	0.034
	Less than 3 days	14 (35.9)	25 (64.1)	
	More than 3 days	12 (35.3)	22 (64.7)	
Received plasma during convalescence	No	354 (50.4)	348 (49.6)	0.056
	Yes	2 (20.0)	8 (80.0)	
Care unit	Home isolation	298 (53.1)	263 (46.9)	0.004
	Home isolation referred to hospital	25 (34.2)	48 (65.8)	
	Hospitalization	33 (42.3)	45 (57.7)	
Received intensive care	No	355 (50.3)	351 (49.7)	0.124^
	Less than 7 days	0 (0)	4 (100)	
	More than 7 days	1 (50.0)	1 (50.0)	
Depression in 60 days	No	281 (48.4)	299 (51.6)	0.083
	Yes	75 (56.8)	57 (43.2)	

^Fischer’s exact test. Other categorical variables were tested with χ^2^ test. All continuous data were tested with Mann–Whitney test.

Considering that symptoms and treatment may have collinearity with disease severity, we subjected these variables to bivariate analysis with the duration of shortness of breath representing the severity of COVID in the clinical setting. Significant correlations and associations were found between shortness of breath and other symptoms, medication, and treatment (S2 Table in S1 File). Hence, we could assume that shortness of breath could represent disease severity.

Table 2 depicts a significant association between several treatments and the prescription of favipiravir, indicating that favipiravir, other medications, and treatments were packaged together. Therefore, other medications and treatments may have a competing effect with favipiravir on post-COVID depression. Thus, we performed mediation and moderator analyses based on the proposed theories of direct and indirect interaction (S3 Table in S1 File). We found that the prescription of antipyretics, azithromycin, the unit of care, and oxygen supplementation had a significant indirect effect on post-COVID depression (p < 0.05) through shortness of breath as a mediator, with cough medication showing a direct effect on depression (S3 Table in S1 File). Furthermore, we observed a significant conditional effect in moderator analysis when assessing the effect of favipiravir on depression, including living alone, sex, and history of hypersensitivity (S3 Table in S1 File).

### Effect of favipiravir

Given that propensity score matching could not control the difference in several variables between groups, these significant factors were subjected to rigorous statistical analysis and were carefully selected as potential confounders in the final analysis. We considered the Cox regression test to identify the effect of favipiravir on depression and adjusted for other confounders and their possible interactions. Given that the initiation of favipiravir varied across individuals, we created a medication-to-event variable, which was the difference between the onset-to-event day and the days of receiving favipiravir as the time-to-event variable. Table 3 shows the effect of favipiravir on depression.

**Table 3:** Effect of favipiravir on post-COVID depression.

Variables	B	SE	df	Sig.	Exp(B)	95.0% CI for Exp(B)	
						Lower	Upper
Age	−.002	.009	1	.786	.998	.980	1.015
Male (Female as reference)	.115	.188	1	.541	1.122	.776	1.622
Living alone	.352	.186	1	.059	1.422	.987	2.048
Positive history of allergy/hypersensitivity	−.279	.330	1	.398	.757	.396	1.444
Duration of shortness of breath	.067	.057	1	.238	1.069	.957	1.195
Receiving favipiravir	−.718	.186	1	**.000**	**.488**	**.339**	**.701**
Not taking antipyretic (ref)			4	.241			
*No fever*	−.653	.633	1	.302	.520	.151	1.799
*Taken <24 h after onset*	−.063	.245	1	.798	.939	.581	1.517
*Taken between 24–72 h*	.090	.298	1	.763	1.094	.610	1.963
*Taken >72 h*	.637	.385	1	.098	1.891	.889	4.020
Not taking anti-inflammatories (ref)			3	.442			
*Taken <24 h after onset*	.203	.223	1	.363	1.225	.791	1.897
*Taken between 24–72 h*	.358	.294	1	.224	1.430	.804	2.544
*Taken >72 h*	.394	.331	1	.233	1.483	.776	2.836
Not taking azithromycin (ref)			3	.003			
*Taken <24 h after onset*	1.036	.307	1	.001	2.819	1.544	5.147
*Taken between 24–72 h*	.940	.309	1	.002	2.560	1.396	4.694
*Taken >72 h*	.351	.387	1	.365	1.420	.665	3.033
Home isolation (ref)			2	.604			
*Home + hospital isolation*	.064	.282	1	.819	1.067	.613	1.855
*Hospital isolation only*	−.265	.307	1	.389	.768	.420	1.402
Did not receive oxygen supplementation			2	.002			
Less than 3 days	.628	.329	1	.057	1.874	.983	3.574
More than 3 days	1.133	.348	1	.001	3.105	1.570	6.141

Favipiravir administration was associated with post-COVID depression (aOR 0.488, 95% CI 0.339–0.701 p < 0.001). Interestingly, we found that the prescription of azithromycin was a significant risk for post-COVID depression. Furthermore, those who received oxygen supplementation for more than 3 days were associated with post-COVID depression in contrast to those who did not.

**Fig 3:**
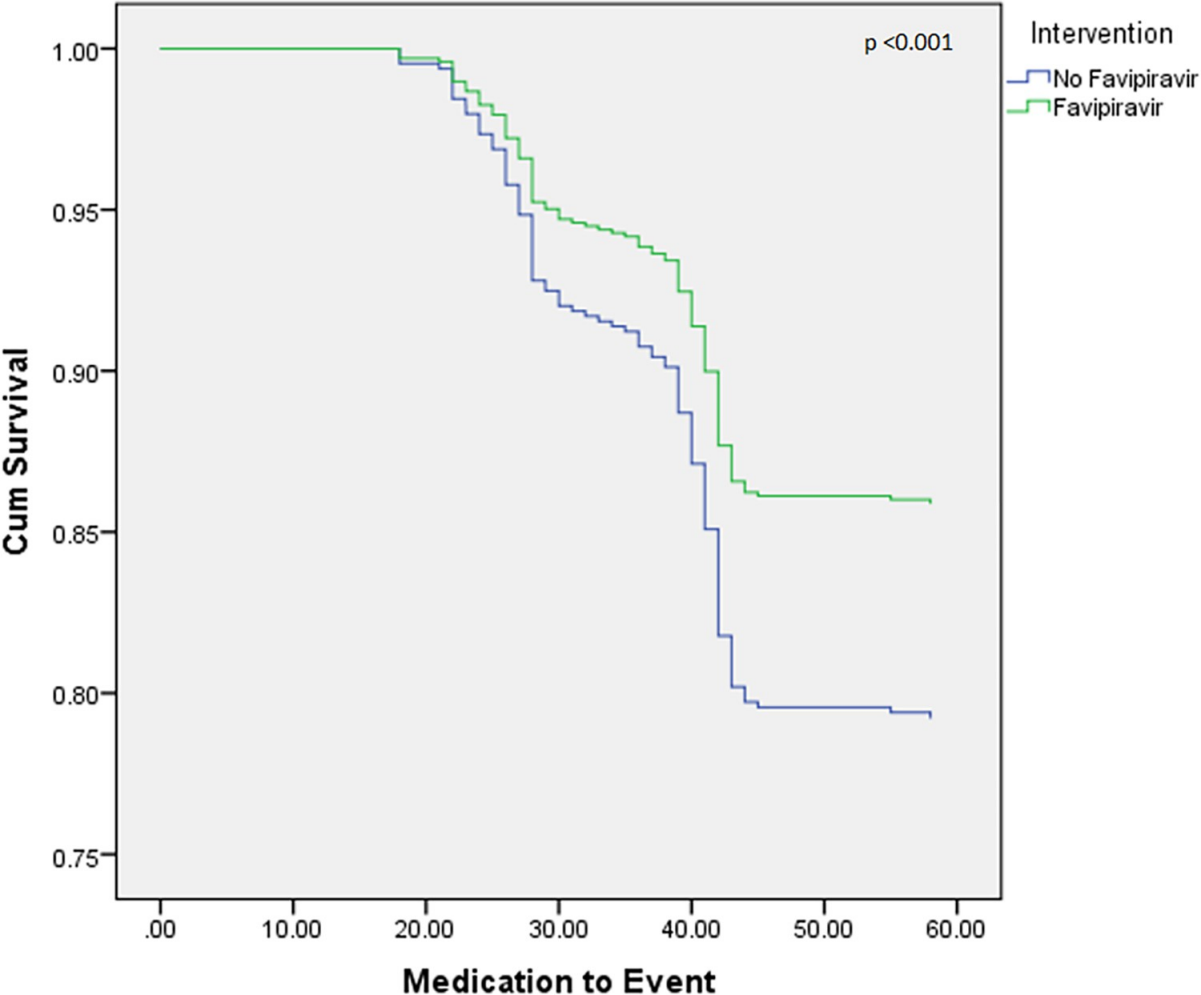
Kaplan–Meier curve of intervention and depression events.

In terms of symptom-specific effects, favipiravir did not affect sleep disorders (S7 Table in S1 File), concentration (S8 Table in S1 File) and fatigue (S9 Table in S1 File). The comparison of participants with no depressive mood + loss of mood for several days versus those who experienced depressive mood for at least more than half the day and nearly every day revealed the significant protective effect of favipiravir (aOR 0.378, 95% CI 0.195–0.731 p = 0.004) (S6 Table in S1 File). This significant protective effect was also seen in the loss of interest/anhedonia (aOR 0.543, 95% CI 0.326–0.906 p = 0.019). However, solitary living was associated with anhedonia (aOR 2.253, 95% CI 1.329–3.818) (S5 Table in S1 File). Fig 3 demonstrates the difference in cumulative survival between the two groups wherein the favipiravir group showed superiority over the nonreceiver group.

## Discussion

### General findings

Although all cases were considered as having mild depression on the basis of the PHQ-9 cutoff, the number of cases of depression in this study was higher than in the global meta-analysis (18.5% versus 12%). The subset of the data appropriately represented the distribution of cases in Indonesia [37], and this study addressed the critical finding that favipiravir exerted a significant effect on preventing depression after COVID. Furthermore, psychosocial factors had a possible role in the development of post-COVID depression, wherein people who lived alone were prone to loss of interest. This study involved various types of patients with different comorbidities, clinical symptoms, and treatments in Indonesia. However, the data were self-reported. In terms of symptom-specific moods, the effect of favipiravir varied across symptoms and different levels of analysis, wherein depressive mood and anhedonia were lower in favipiravir recipients.

### Effect of the study design and data collection

In contrast to that in other prospective longitudinal studies, in this study, we started data analysis by extracting a subset of data from the primary cohort, which consisted of clinical and nonclinical data, for baseline information. Our data source was patients, particularly those who could only access the home isolation program, which other clinical studies may not consider. The telemedicine program has been integrated into COVID services either from healthcare services or health applications provided by third parties, and plays an essential role in reducing the health service burden. Hence, this approach could increase the generalizability of the findings.

The cohort timeline covered only the second wave of COVID, which was dominated by the delta variant. We did not expand the study through the omicron wave, which started in early February 2022, due to the time constraint. We were also unable to assess the alpha and beta periods (before July 2021) given that the outcome measurement would be affected by recall bias. Hence, we cannot accommodate the possible effect of different variants on depression.

We extracted the data by using propensity scores because we could not purposively recruit and randomize the participants for this study. Despite having a rigorous propensity technique with the smallest difference, we still faced difficulties in reducing the heterogeneity between the two groups on the basis of the selected propensity variables (age, sex, hypersensitivity, and island). The underlying reason was that favipiravir is prescribed for a particular condition. First, studies have shown that favipiravir might induce hypersensitive reactions and other adverse events at a low percentage [38]. As a result, no prescription would be made for hypersensitive individuals. Second, men and the elderly are associated with severe diseases. Therefore, the use of favipiravir is preferred in these groups. Third, the distribution of favipiravir is uneven, and oseltamivir is preferred in other regions except Java [39]. Nevertheless, we executed bivariate, moderator, and mediator analyses to explore the possible interactions and confounding effects to overcome the heterogeneity originating from propensity allocation.

Self-reported information is always prone to either recall or procedural bias. First, the reporting of the results in clinical settings was delayed particularly during the surge of COVID cases. Second, drugs were prescribed after diagnosis instead of onset, and delayed treatment was inevitable. However, prior to diagnosis, several medications (antipyretics and multivitamins) could be purchased over the counter. Third, the definition of isolation release and recovery status differed in accordance with severity and physician discretion. These factors should be accommodated because every individual would have time-varying variables. Given that we could estimate the medication-to-event time, Cox regression analysis was more appropriate than repeated measurement analysis.

An issue regarding the medication dosage and adherence that may affect the efficacy of favipiravir and the interaction of other drugs also existed. The dosage of favipiravir in Indonesia’s guideline is in line with the minimum recommended dose. However, the number of pills for loading and maintenance doses was too high (eight tablets once for loading doses) to be taken at once. Hence, ruling out nonadherence to medication, particularly in asymptomatic and mild cases, remains difficult.

Assessing and interpreting the outcome was another issue. Although the baseline information was collected retrospectively, we performed a prospective longitudinal follow-up every 2 weeks because PHQ-9 describes the situation over the past 2 weeks and identified the first day of depression onset. PHQ-9 could accommodate feelings of anhedonia (or loss of interest) and somatic disorders, including fatigue and the deterioration of concentration and sleep. Furthermore, we compared depressive symptoms in accordance with the DSM-5 criteria to strengthen the outcome interpretation. However, a structured clinical interview, which is preferred for confirmation, was unfeasible.

We did not extend the follow-up period because of the low response rate in the following weeks. A study pointed out that the association between depression 30 days after recovery was stronger than that after 120 days postrecovery [40]. Therefore, 60 days of follow-up was sufficient, and a longer assessment was unnecessary.

To sum up, we recognized and handled several issues with methodologies, including data collection, data collection, and medication adherence, that could affect the interpretation of the results. Nevertheless, several issues should still be taken into account.

### Favipiravir and depression: A plausible explanation

In accordance with the analysis results, addressing the significant effect of the severity of the disease on the occurrence of post-COVID depression before the impact of favipiravir is interpreted is imperative. Shortness of breath is one of the essential clinical features of COVID and mainly considered to represent the severity of the disease [41]. In our final model, a longer duration of shortness of breath was not associated with depression. However, oxygen supplementation was strongly associated with depression. Oxygen is given following persistent desaturation, and the manifestation of desaturation may not always appear as shortness of breath. In patients with COVID-19, prolonged desaturation may affect the nervous system. Favipiravir was preferred in some patients with apparent clinical symptoms and has been proven to impact depression significantly. Our first assumption is that favipiravir reduces viral replication and prevents subsequent injury to the CNS, particularly in the area responsible for cognitive function and behavior. This phenomenon could be the principal mechanism through which favipiravir can prevent depression. A longitudinal study focusing on brain metabolic patterns in COVID-19-related encephalopathy reported a hypometabolic pattern in the frontal cortex, anterior cingulate, insula, and caudate nucleus that mainly improved after 6 months. The participants’ clinical symptoms showed predominant cognitive and behavioral frontal disorders linked to these specific brain regions [42]. Hence, favipiravir administration was assumed to prevent the injury of these depression-related regions in the brains of patients with COVID-19. Mediator analysis demonstrated that favipiravir exerted an indirect effect on depression mediated by shortness of breath (S3 Table in S1 File). Furthermore, shortness of breath was strongly associated with oxygen supplementation (S2 Table in S1 File). Hence, we concluded that favipiravir indirectly affects depression by preventing CNS injury due to hypoxia and prolonged desaturation in depression-associated brain regions. One thing to note is that we were unable to prove the indirect effect of favipiravir and depression through inhibition of viral replication because we did not measure viral levels.

The severity of the disease was also indirectly associated with depression through the alteration of the neurotransmitter pathway. Tryptophan absorption and metabolism in patients with COVID-19 reduced. Our cohort presented a positive correlation between diarrhea and shortness of breath, as shown in S2 Table in S1 File. Therefore, we assumed that the gastrointestinal disturbances observed in our participants was linearly related to the severity of the disease. Our findings suggested that favipiravir may indirectly reduce depression by inhibiting viral replication, thus preventing the direct neurological and gastrointestinal injuries that are vital for tryptophan absorption. However, the indirect analysis following this theory did not support this assumption (S3 Table in S1 File).

The question of how favipiravir itself directly affects the CNS, including regarding neurotransmitters, remains. However, the penetration of favipiravir into the BBB is low, and animal studies demonstrated that the dose of favipiravir must be increased to achieve an in vivo antiviral effect on the CNS [43]. Furthermore, the site of action of favipiravir is at the RdRP of the RNA virus, which does not affect human cells.

Given that favipiravir is packaged with other medications in accordance with guidelines, the question whether other medicines affect depression remained. In this study, we performed the mediator analysis of other medications. Vitamin D was linked to a lower level of depression [44] and was recently prescribed for patients with COVID-19 given that the level of vitamin D was lower in this group [45]. However, this study did not find any significant direct or indirect effect of vitamin D on depression (S3 Table in S1 File). Zinc has been introduced into the COVID-19 treatment protocol since the early phase and has been shown to activate a mechanism in the CNS, particularly the hippocampus, amygdala, and brain cortex, with glutamatergic neurons. This mechanism could explain the inverse correlation between zinc supplementation and depression in various studies [46]. Similar to vitamin D, zinc also revealed a nonsignificant impact on depression. However, considering their possible mediatory effect, the significant effect of antipyretics, azythromicin, and cough medication were still taken into consideration. Despite the presence of these medications as adjusting factors, favipiravir remained beneficial in reducing post-COVID depression.

### Post-COVID depression might not only be due to neurological changes

Depression and mental health disorders during the post-COVID period may not be due only to neurological changes but also psychosocial factors. Aside from demographic characteristics, self-isolation and the presence of sequelae symptoms played a role in post-COVID depression. In addition, psychosocial factors may execute different roles at different time points and influence the actual effect of favipiravir. Hence, conducting Cox regression analysis to identify the impact of psychosocial factors across time was appropriate.

Solitary living was associated with the higher loss of interest. Stricter home isolation and difficulties obtaining supportive care, particularly in addressing persistent symptoms, could lead to ongoing depression. We could assume that depression symptoms were not purely due to neurological changes. A limitation of this study is its inability to perform robust psychosocial assessment through a clinical interview to obtain additional reliable information. Nevertheless, by applying advanced statistical analysis to accommodate general psychosocial factors, we were able to identify the effect of favipiravir in the prevention of post-COVID depression.

### Strengths and limitations

This study involved all severity levels, comorbidities, and various treatments of patients with COVID-19 from every part of Indonesia. Nevertheless, we should be aware of the recall bias and quality of the retrospective baseline. We applied matching to provide an unbiased cohort and rigorous statistical analysis to demonstrate the actual effect of favipiravir. Nevertheless, strengthening the results of this study by conducting biomarker assessments, as well as serotonin and psychosocial investigations, on all participants is vital.

### Clinical and public health implications

Although additional studies with robust methodology are needed, we recommend that patients with COVID-19 should take the prescribed favipiravir regardless of the dispensing time. Post-COVID depression is mainly prevented through an indirect mechanism based on the prevention of CNS injury. These findings could be a convincing piece of evidence for enhancing adherence to favipiravir considering that many patients feel reluctant to take multiple pills at higher daily frequencies and are unaware of the long-term benefits of taking favipiravir. Furthermore, healthcare providers must ensure the availability of mental health services and integrate such services into current guidelines because the core of depression in COVID consists of multiple factors, including psychosocial factors. Expanding depression screening to patients with COVID-19, particularly those who were hospitalized and requiring outpatient care, is necessary to enhance rehabilitation therapy.

## Supporting information

S1 File (XLSX)
